# Lung Metastasis in a Case of Recurrent Poorly Differentiated Leiomyosarcoma of the Bartholin Gland: A Case Report and Review of the Literature

**DOI:** 10.7759/cureus.550

**Published:** 2016-03-31

**Authors:** Fatimah Alnafisah, Joanne Alfieri

**Affiliations:** 1 Obstetrics & Gynecology Department, King Saud Hospital, Saudi Arabia; 2 Department of Radiation Oncology, McGill University Health Centre

**Keywords:** leiomyosarcoma, bartholin gland, vulvar sarcoma

## Abstract

Vulvar neoplasms represent four percent of all gynecological cancers. While most cases of vulvar neoplasms are benign, two percent of patients present with malignant disease. We present the case of a 37-year-old premenopausal female who presented to an outside institution with a lump in her left vulva, which had progressively enlarged to the size of an egg. A wide local excision of the left vulva was performed, and the pathology revealed a high-grade sarcoma, not otherwise specified (NOS), with negative margins. Imaging showed enlarged bilateral external iliac lymph nodes, likely metastatic. After discussion at a multidisciplinary gynecology oncology tumor board, she was treated with gemcitabine/docetaxel chemotherapy, followed by a left inguinal lymph node dissection and a left radical vulvectomy after being referred to our centre. The final pathology at that time showed a residual sarcoma of 3.5 mm in the left vulva with no lympho-vascular invasion (LVI) and negative margins, with the closest, laterally, at 2 mm. A total of three lymph nodes were negative. She received additional chemotherapy postoperatively. Approximately one year later, she returned to her gynecologist with a 1 cm mass on the left vulva. She underwent a left hemi-vulvectomy and lymph node dissection, and pathology confirmed the presence of a high-grade sarcoma with close margins. She received adjuvant radiotherapy. Three months later, she presented with persistent cough and pneumonia. Imaging revealed a 10 cm lung mass, which was believed to be metastasis from the vulva. This was confirmed with biopsy and was completely resected.

Any mass in the Bartholin gland area should be investigated carefully. Poorly differentiated vulvar leiomyosarcoma in the Bartholin gland can recur locally but may also lead to distant metastasis. Despite surgical and systemic treatment, as well as adjuvant radiation, the tumor recurred. Due to the rarity of this condition, there are no clear recommendations for treatment of this disease. To our knowledge, this is the first report of vulvar leiomyosarcoma of the Bartholin gland with metastasis to the lung.

## Introduction

Sarcomas are uncommon malignant tumors of mesenchymal origin that comprise two percent of all cancers in humans [1]. Ninety percent of sarcomas of the female reproductive system are uterine sarcomas. Sarcomas represent approximately one to three percent of all tumors in the area of the vulva [1]. The histological types of vulvar sarcomas are leiomyosarcomas, epithelioid sarcomas, rhabdomyosarcomas, angiosarcomas, malignant peripheral nerve sheath tumors, malignant fibrous histiocytomas, and sometimes aggressive angiomyxomas [1]. Leiomyosarcoma is the commonest among these histological types [1-2]. It is believed that the labia majora are the most common site of vulvar leiomyosarcoma followed by the Bartholin gland, clitoris, and labium minus [3]. In general, vulvar sarcomas usually present as rapid-growing nodules with frequent recurrences, which are characterized by a high chance of metastasis, and an increased mortality rate. These tumors may arise from smooth muscles, blood vessels, rough ligaments, and erector-pili muscles [1-2, 4].

There are very few cases of vulvar leiomyosarcomas reported as small case series or as case reports. This case is unique in that there were no distant metastases in previously reported cases of leiomyosarcoma of the Bartholin gland [2]. Informed consent was obtained from the patient for this study.

## Case presentation

A 37-year-old premenopausal female (gravida 6, para 3, aborta 3), ex-smoker whose past medical history was only significant for hyperlipidemia, presented to a community hospital in August of 2012 with a lump in her left vulva. What began as a pea-sized mass had rapidly enlarged to the size of an egg. She had a wide local excision of the left vulva with the pathology revealing a high-grade sarcoma, NOS. The tumor measured 5.0 x 3.9 x 2.9 cm, and it displayed ulceration of the surface epithelium, with no definite lymphovascular invasion (LVI), strong expression of estrogen and progesterone receptors, and focal positivity for smooth muscle markers compatible with smooth muscle differentiation (i.e., leiomyosarcoma) with negative margins. Endometrial biopsy and Pap test were also performed at that time and showed no evidence of malignancy. She was then referred to our institution where staging computed tomography (CT) scan of the chest, abdomen, and pelvis revealed enlarged bilateral external iliac lymph nodes that were likely metastatic. A positron emission tomography (PET)/CT scan performed at that time only showed some nonspecific activity in the vaginal/vulvar region likely related to her recent surgery. After further discussion at the tumor board, she received three cycles of gemcitabine/docetaxel chemotherapy prior to undergoing completion left radical vulvectomy and left inguinal lymph node dissection. The pathology from this second surgery showed residual sarcoma measuring 3.5 mm in the left vulva, no LVI and negative margins, with the closest laterally at 2 mm. Three lymph nodes were found to be negative. She received three more cycles of gemcitabine/docetaxel postoperatively.

**Figure 1:**
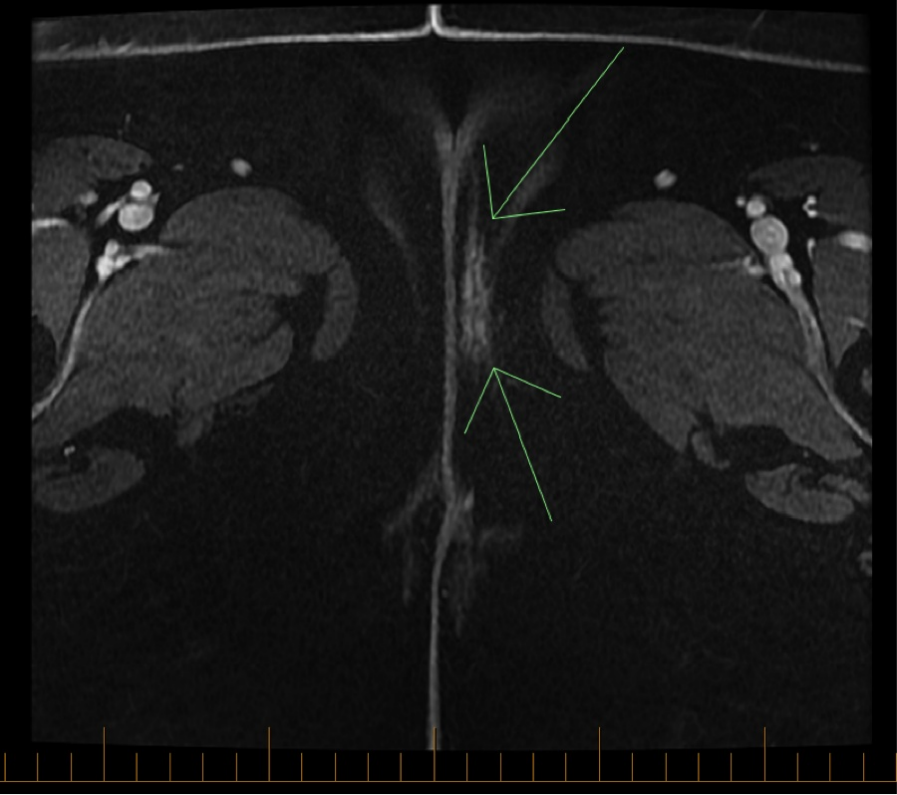
Axial MRI of the pelvis showing an abnormal enhancement in the left vulvar area measuring 3.7 cm in greatest dimension, representing tumor recurrence.

Fifteen months later, a 1 cm mass on her left vulva was discovered in the same location as the original tumor at a follow-up visit. A magnetic resonance imaging (MRI) of the pelvis showed an abnormal enhancement in the left vulvar area measuring 3.7 cm in greatest dimension (as seen in Figure 1), consistent with recurrent vulvar disease. The bilateral inguinal and bilateral pelvic lymph nodes were stable; however there was a new 7 mm left inferior epigastric lymph node. On PET/CT scan, a new distinct focal uptake was noted lateral to the vaginal wall on the left side with a standard uptake value (SUV) of six for which neoplastic disease was in the differential. No distant metastases were present.

The patient then underwent a left hemivulvectomy, and a left external iliac, left inferior epigastric, and a left obturator lymph node excision. In the vulva, there was a small focus of residual high-grade sarcoma measuring 1.5 mm, with tumor at 2.0 mm from lateral margin. There was no LVI noted, and all 14 lymph nodes resected were negative.

Two months after the surgery, the patient underwent high dose rate interstitial brachytherapy and external beam radiation therapy to the vulva. The dose of the interstitial brachytherapy was 15 Gy in 5 fractions, delivered b.i.d. with atleast a 6-hour interval for three consecutive days. Six metal needles were placed guided by a perineal template. The volume to cover was delineated on CT with fusion of the diagnostic MRI. This was followed by 45 Gy in 25 fractions (five days per week, five consecutive weeks) of external beam radiotherapy using intensity modulated radiation therapy. The patient required a four-week treatment break between the brachytherapy and external beam treatment due to development of an ulcer on the left lateral vaginal wall. The clinical target volume included the scar and entire tumor bed with a 1 cm margin and the remainder of the left hemivulva. The planning target volume (PTV) included the clinical target volume (CTV) with a 1 cm margin.

On the first follow-up after treatment completion, the patient was very well, with no pain or local irritation. Upon examination, the 1 cm healing ulcer was still visible. There was no evidence of disease.

**Figure 2:**
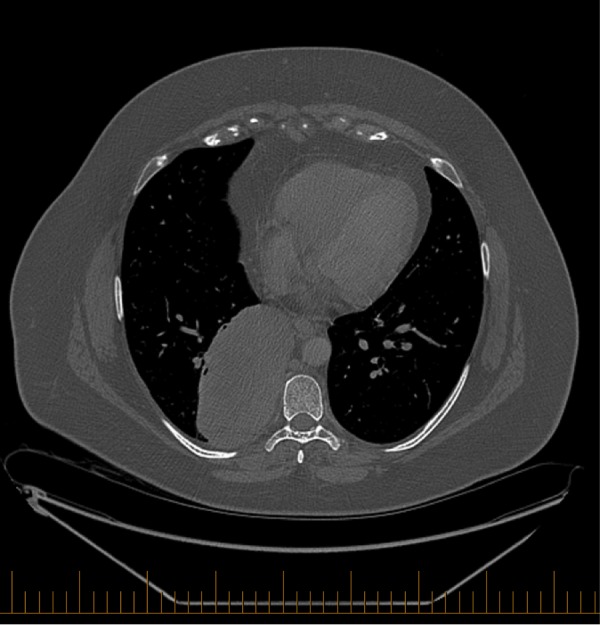
Axial CT scan of the chest showing a 10-cm paravertebral, subpleural thoracic mass.

Three months later, the patient was asymptomatic in the vulva, with the ulcer on the left vulva still present and stable in size. She did admit to having a cough and to have suffered from pneumonia several weeks prior, which was treated with antibiotics. She also experienced pain in the lower thoracic spine region when lying on her right side, and in the upper abdomen or lower chest when coughing. She was otherwise well, with no constitutional symptoms. Her appetite and energy were normal, and her weight was stable. The patient’s general physical examination was normal. On pelvic MRI, only postoperative changes were visible at the level of the vulva. However, on CT scan of the chest, a large, 10 cm paravertebral, subpleural mass (as seen in Figure 2) was found, occupying the posteromedial aspect of the right lower lobe.

A biopsy was done confirming metastatic leiomyosarcoma consistent with the vulvar primary. Restaging PET scan was done confirming the single lung metastasis. As this was deemed the only site of disease, the patient underwent a right lower lobectomy. On pathology, a tumor measuring 18.0 x 14.0 x 6.5 cm was identified in the posterolateral aspect of the right lower lobe. It was well-circumscribed, multilobulated, and it appeared to have multiple areas of necrosis and hemorrhage. Examination of the adjacent lung parenchyma appeared unremarkable. The vascular, bronchial, and parenchymal margins were uninvolved. Immunohistochemistry supported the diagnosis of metastatic leiomyosarcoma. The patient went on to receive cisplatin/adriamycin chemotherapy. A follow-up and repeat PET scan two months after her surgery showed no evidence of local or distant disease.

## Discussion

We present a case of locally recurrent vulvar leiomyosarcoma of the Bartholin gland with distant metastasis to the lung. Smooth muscle neoplasms of the vulva are rare. The majority are seen in women of middle and older age (age range, 20 to 68 years old), and the average age of clinical presentation is approximately 30–40 years old [1]. The youngest age reported was 14 years of age [5]. Most of the cases reported in the literature are from Western countries, which may point to lifestyle or genetic factors related to these tumors [1]. The cause of these tumors is unclear. It has been hypothesized that chronic inflammation is an important component of tumorigenesis [1]. In fact, vulvar leiomyosarcoma in patients with longstanding lichen sclerosus has been reported [6]

Leiomyoma and leiomyosarcoma of the Bartholin gland are often clinically misdiagnosed as Bartholin gland cysts or abscesses [4]. Leiomyosarcomas of the Bartholin gland are rare, and because of the anatomic location of the tumor, it is usually unnoticeable to the patient until the mass is large or causes symptoms such as pain, pruritus, and erythema. These factors lead to a delay in the diagnosis and impede early intervention, resulting in advanced presentations and a worse prognosis. Biopsy is highly recommended if a mass of the Bartholin gland is firm or solid on palpation, and if it is ulcerated or found in a slightly different location than the usual Bartholin gland cyst [4,7]. Early diagnosis and treatment are important because these smooth muscle neoplasms have high risk of recurrence, and patients may require adjuvant radiotherapy in addition to adequate surgical excision [4].

In 1996, Nielsen et al. proposed criteria to differentiate between leiomyosarcoma and leiomyoma: 1) The mass should be more than 5 cm in diameter; 2) There should be the presence of an infiltrative margin; 3) There are more than five mitotic figures per 10 HPF; and 4) There is moderate to severe cytologic atypia. Tumors with three or more of these features should be considered sarcomas and those that have only one can be diagnosed as leiomyoma, while two of these characteristics can be considered as atypical leiomyoma [8]. In the presenting case, the pathology showed a high-grade sarcoma, NOS. The tumor measurements were of 5 cm in greatest dimension, and it displayed ulceration of the surface epithelium, and focal positivity for smooth muscle markers compatible with smooth muscle differentiation (i.e., leiomyosarcoma). The tumor had characteristics of a high-grade sarcoma and exhibited small cell-like features. With this morphology, the possibilities of extraskeletal Ewing’s sarcoma, alveolar rhabdomyosarcoma, and synovial sarcoma were considered in the differential diagnosis. The three possibilities were excluded by molecular studies. Immunohistochemistry results showed the tumor was strongly and diffusely positive for vimentin, estrogen, and progesterone receptors; CD99 and NSE were positive in most of the tumor cells; Desmin and MSA were focally positive; and rare tumor cells were positive for S-100 and HMB45. The tumor was completely negative for Keratin AE1/AE3, CK8-18, EMA, MelanA, GFAP, C-Kit, CD31, CD34, chromogranin, synaptophysin, and MDM2. The tumor was classified as high-grade sarcoma, NOS, The presence of strong estrogen receptor (ER) and progesterone receptor (PR) expression and focal positivity for Desmin and MSA in this clinical context was felt to be compatible with an unusual variant of leiomyosarcoma. Immunohistochemical evaluation is important to confirm the diagnosis of vulvar leiomyosarcoma in order to dictate subsequent management [9].

In 2003, Ulutin et al. reviewed 453 cases with vulvar malignancy from 1977-1997 [3]. Seven patients had vulvar sarcoma (1.54%). Three cases had leiomyosarcoma, two patients had fibrosarcoma, and one case had epithelioid sarcoma. One patient was excluded from the study because the histology was confirmed to be alveolar rhabdomyosarcoma in a very young patient. The three cases of vulvar leiomyosarcoma all were located in the labia majora. One of the three patients presented with a mass measuring 7 cm, with more than 3 mm of invasion, treated with radical vulvectomy and groin lymph node dissection with adjuvant radiotherapy. The size of the tumors in the other two patients was 3 cm and 3.5 cm, with less than 3 mm invasion, and treated with simple vulvectomy and radical vulvectomy respectively, without adjuvant radiotherapy [3]. In their study, none of the leiomyosarcoma cases had metastasized.

Aartsen and Albus-Lutter reviewed the history of 47 patients with vulvar sarcoma, which included 25 cases of leiomyosarcoma [10]. Seventeen of them had at least one local recurrence (68%), and 13 had more than one recurrence (52%). Eight patients developed disseminated disease, five to lung (20%), one to liver (4%), and three to other distant sites (12%) [10]. None of the eight cases had primaries in the anatomical location of the Bartholin gland.

In 2009, Gonzalez-Bugatto et al. published a review of seven cases of vulvar leiomyosarcoma of the Bartholin gland [2]. During the follow-up period (13 months to 24 years), none of the patients developed lymph node or distant metastases, and all of the patients were alive without evidence of disease. Four of the seven cases had local recurrence [2], a common event in leiomyosarcoma.

These tumors have a very low incidence, and the literature shows a wide variety of treatment plans limited to a small number of patients [2]. Wide local excision with at least a 2-cm clear margin confirmed by pathological evaluation is highly recommended [2,4]. The risk of local recurrence is high in patients with inadequate resection margins [2,10]. For small, well-circumscribed tumors with adequate clear margins, wide local excision is sufficient [2]. For low-grade sarcomas with free surgical margins, the patients need close observation and adjuvant radiotherapy may not required. In cases with residual sarcoma, close surgical margins, or with high-grade tumors, adjuvant radiotherapy is indicated [3]. In the case of local recurrence, wide local excision with adjuvant radiotherapy is the treatment of choice. The role of chemotherapy is not clearly defined [2,4]. Chemotherapy has been reported to produce regression of lung metastasis in vulvar sarcoma [2,10].

In this particular case, the patient was treated first with wide local excision. After the incidental finding of leiomyosarcoma, referral to our centre prompted staging studies which showed enlargement of the bilateral external iliac lymph nodes. She had received preoperative chemotherapy before undergoing left radical vulvectomy and left inguinal lymph node dissection, followed by additional chemotherapy. Fifteen months later, the tumor recurred locally and the patient was treated with left hemivulvectomy and lymph node dissection followed by interstitial brachytherapy and external beam radiotherapy. Three months later, the patient developed lung metastasis, treated by lobectomy and adjuvant chemotherapy. The patient remains disease free two months from completion of therapy.

## Conclusions

To our knowledge, this is the first case of vulvar leiomyosarcoma of the Bartholin gland with lung metastasis to be reported. A review of the literature shows few cases of leiomyosarcoma of the Bartholin gland, and none of these cases have distant metastases. Cases with multiple local recurrences and high-grade tumors generally have a poor prognosis [2]. Early diagnosis and treatment are important because these sarcomas have a high risk of recurrence. Any vulvar lesion should be investigated carefully, and biopsy is recommended. Despite locoregional control achieved through surgical intervention and adjuvant radiotherapy, the tumor recurred distantly in our patient. The most optimal treatment regimen remains unknown. There are no standardized guidelines or clear recommendations in the management of this type of sarcoma. Further studies examining different systemic treatments are required to elucidate the best treatment strategy.
